# Reducing fresh full term intrapartum stillbirths through leadership and accountability in a low-resource setting, Mpilo Central Hospital, Bulawayo, Zimbabwe

**DOI:** 10.1186/s13104-017-2567-z

**Published:** 2017-07-06

**Authors:** Solwayo Ngwenya

**Affiliations:** 1 Department of Obstetrics & Gynaecology, Mpilo Central Hospital, Vera Road, Mzilikazi, P.O. Box 2096, Bulawayo, Matabeleland Zimbabwe; 2 Department of Obstetrics & Gynaecology, Royal Women’s Clinic, 52A Cecil Avenue, Hillside, Bulawayo, Matabeleland Zimbabwe; 3 National University of Science and Technology, Medical School, Bulawayo, Matabeleland Zimbabwe

**Keywords:** Fresh full term intrapartum stillbirths, Leadership, Accountability, Perinatal outcomes, Low-resource settings, Mpilo Central Hospital

## Abstract

**Background:**

Stillbirths are distressing to the parents and healthcare workers. Globally large numbers of babies are stillborn. A number of strategies have been implemented to try and reduce stillbirths worldwide. The objective of this study was to assess the impact of leadership and accountability changes on reducing full term intrapartum stillbirths.

**Methods:**

Leadership and accountability changes were implemented in January 2016. This retrospective cohort study was carried out to assess the impact of the changes on fresh full term intrapartum stillbirths covering the period 6 months prior to the implementation date and 12 months after the implementation date. The changes included leadership and accountability. Fresh full term stillbirths (>37 weeks gestation) occurring during the intrapartum stage of labour were analysed to see if there would be any reduction in numbers after the measures were put in place.

**Results:**

There was a reduction in the number of fresh full term intrapartum stillbirths after the introduction of the measures. There was a statistical difference before and after implementation of the changes, 50% vs 0%, *P* = 0.025. There was a reduction in the time it took to perform an emergency caesarean section from a mean of 30 to 15 min by the end of the study, a 50% reduction.

**Conclusions:**

Clear and consistent clinical leadership and accountability can help in the global attempts to reduce stillbirth figures. Simple measures can contribute to improving perinatal outcomes.

## Background

Mpilo Central Hospital is located in Bulawayo and deliveries 9000 deliveries per year. Bulawayo is the second largest city in Zimbabwe after the capital city Harare, with a population of 653,337 as of the 2012 census [1]. It is located in Matabeleland, 439 km southwest of Harare, on the way to Victoria Falls.

Stillbirths remain a global headache. Annually 2.6 million stillbirths occur worldwide, 98% in developing countries [2]. Each year 1.02 million intrapartum stillbirths and 904,000 intrapartum-related neonatal deaths occur [3]. Pregnancy loss leads to long-standing parental depression and related problems [4]. The low proportion of intrapartum stillbirth in high-income countries suggests that intrapartum stillbirth are largely preventable with quality intrapartum care including prompt recognition and management of intrapartum complications [5]. Substandard care contributes 20–30% of cases [6]. The perinatal mortality rate for Zimbabwe as of 2014 was 39 per 1000 live births [7].

There are no similar studies in the literature specifically on reducing stillbirths through leadership and accountability done in low-resource settings. This study aims document if simple measures could help reduce preventable perinatal deaths especially where resources are limited.

## Methods

This was a retrospective cohort study carried out at Mpilo Central Hospital, a tertiary teaching referral government hospital in a low-resource setting. Information was obtained from the birth registers/case notes in labour ward/theatres covering the period 1 July, 2015 to 31st December, 2016. The changes included redeploying experienced midwives back to labour ward (from other wards where they had been inappropriately deployed), registrars were put on resident on-call and a second theatre was brought back into function. The time of decision for emergency caesarean section to cutting time was monitored. Every case note of fresh full term intrapartum stillbirth was scrutinised. Health workers were made accountable by writing statements. Fresh stillbirths that were already dead at the time of admission were excluded from the study. The stillbirths from congenital abnormalities and preterm deliveries were also excluded from the study. Only fresh full term intrapartum stillbirths (>37 weeks gestation) occurring during the intrapartum stage of labour were analysed. The SPSS Version 21(IBM Corp., Armonk, NY, USA) statistical tool was used to calculate the mean and standard deviation (SD) figures. Simple statistical tests were used on absolute numbers to calculate percentages. Chi-square test was used to calculate *P* values. A *P* value of <0.05 was considered statistically significant.

## Results

Table 1 shows the results of the study. There were 16 fresh full term intrapartum stillbirths in 6 months prior to the implementation of the changes. During this period (July 2015–December 2016), fresh full term intrapartum stillbirths constituted between 40 and 100% of the fresh full term stillbirths at the unit. In the middle of the study (January 2016–October 2016) they were 0–33%. At the end of the study (December 2016), they constituted 0% of the fresh full term stillbirths. There were 3 fresh full term intrapartum stillbirths for 12 months after the changes were made. The other fresh full term stillbirths were those foetuses whose mothers arrived at the unit with the baby already dead. There was a statistical difference (50% vs 0%, *P* = 0.025) between the fresh full term intrapartum stillbirths at the beginning and at the end of the study. The mean time from the time of decision for caesarean section to cutting time was 30 min (SD ±10) at the beginning of the study. At the end of the study it was 10 min (SD ±5). This was a 50% reduction.

**Table 1:** The distribution of total deliveries and fresh stillbirths

Month	Total deliveries	Total FSB	Full term FSB	Other conditions
Before measures				
July 2015	759	4	2 (50%)	2 (50%)
August	720	5	2 (40%)	3 (60%)
September	724	2	2 (100%)	0 (0%)
October	756	3	3 (100%)	0 (0%)
November	702	6	5 (80%)	0 (0%)
December	726	2	2 (100%)	1 (20%)
After measures				
January 2016	735	3	1 (33.3%)	2 (66.7%)
February	763	6	0 (0%)	6 (100%)
March	784	3	0 (0%)	3 (100%)
April	735	4	0 (0%)	4 (100%)
May	767	5	0 (0%)	5 (100%)
June	783	5	1 (20%)	4 (80%)
July	735	1	0 (0%)	1 (100%)
August	798	2	0 (0%)	2 (100%)
September	760	2	0 (0%)	2 (100%)
October	735	4	1 (25%)	3 (75%)
November	711	6	0 (0%)	6 (100%)
December	694	1	0 (0%)	1 (100%)

## Discussion

This study documents the effect of implementation of simple measures in a unit challenged by low resources. There was a reduction in preventable fresh full term intrapartum stillbirths in the unit after the introduction of measures driven by clinical leadership and accountability.

Before the changes were put in place, fresh full term intrapartum stillbirths constituted 40–100% of the cases of fresh full term stillbirths. The changes took some time to show some improvements. By the middle of the study, fresh full term stillbirths constituted 0–33%. Healthcare workers were learning from their mistakes at monthly audit meetings. At the end of the study, fresh full term intrapartum stillbirths were in the figures of 0%. This meant that women arriving at the unit with a live fetus, the chances of a fresh full term stillbirth had significantly reduced (*P* = 0.025). The use of two operating theatres helped improve waiting times for emergency operations by 50%.

There were 16 fresh full term intrapartum stillbirths for the 6 months prior to the changes and there were only 3 fresh full term intrapartum stillbirths for the entire 12 months after the implementation of the changes. There used to be at least 2 fresh full term intrapartum stillbirths per month prior to the measures being put in place to 1 every 4 months at the end of the study.

Intrapartum-related neonatal deaths can be avoided by interventions that prevent intrapartum complications, detect and manage intrapartum problems [8]. Simple, affordable and effective approaches are available for low-resource settings [9]. Audits are associated with improved quality of care [10–12]. Audits provide insight into where systematic deficiencies in clinical care occur and can provide crucial direction for the targeting of interventions [13]. Consistent leadership and accountability can many save lives globally [14]. The five priority areas to change and end preventable deaths by 2030 the stillbirth trend include intentional leadership, increased voice especially of women, implantation of integrated interventions with commensurate investment [15] at local, national and international levels.

This study is very important as it documents simple measures that clinicians can adopt and adapt. These simply measures can help improve perinatal outcomes. Accountability made sure that all staff looking after pregnant women in labour took due care simple they knew that they were being watched and that each poor outcome would be scrutinised.

## Conclusions

Good leadership and accountability can help reduce poor perinatal outcomes. Simple measures like staff redeployments, use of extra operating theatres can improve outcomes. These measures do not cost money and would be appropriate in low-resourced settings.

## Abbreviations

FSB: fresh stillbirth
SD: standard deviation
